# New insights into the Upper Palaeolithic of the Caucasus through the study of personal ornaments. Teeth and bones pendants from Satsurblia and Dzudzuana caves (Imereti, Georgia)

**DOI:** 10.1371/journal.pone.0258974

**Published:** 2021-11-08

**Authors:** José-Miguel Tejero, Guy Bar-Oz, Ofer Bar-Yosef, Tengiz Meshveliani, Nino Jakeli, Zinovi Matskevich, Ron Pinhasi, Anna Belfer-Cohen

**Affiliations:** 1 Department of Evolutionary Anthropology, University of Vienna, Vienna, Austria; 2 Seminari d’Estudis I Recerques Prehistòriques, Universitat de Barcelona, Barcelona, Spain; 3 Zinman Institute of Archaeology, University of Haifa, Haifa, Israel; 4 Department of Anthropology, Harvard University, Cambridge, MA, United States of America; 5 Georgian National Museum, Tbilisi, Georgia; 6 Israel Antiquities Authority, Jerusalem, Israel; 7 Institute of Archaeology, The Hebrew University of Jerusalem, Jerusalem, Israel; Universita degli Studi di Ferrara, ITALY

## Abstract

The region of western Georgia (Imereti) in the Southern Caucasus has been a major geographic corridor for human migrations during the Middle and Upper Paleolithic. Data of recent research and excavations in this region display its importance as a possible route for the dispersal of anatomically modern humans (AMH) into northern Eurasia. Nevertheless, within the local research context, bone-working and personal ornaments have yet contributed but little to the Upper Palaeolithic (UP) regional sequence’s characterization. Here we present an archaeozoological, technological and use-wear study of pendants from two local UP assemblages, originating in the Dzudzuana Cave and Satsurblia Cave. The ornaments were made mostly of perforated teeth, though some specimens were made on bone. Both the manufacturing marks made during preparation and use-wear traces indicate that they were personal ornaments, used as pendants or attached to garments. Detailed comparison between ornament assemblages from northern and southern Caucasus reveal that they are quite similar, supporting the observation of cultural bonds between the two regions, demonstrated previously through lithic techno-typological affinities. Furthermore, our study highlights the importance attributed to red deer (*Cervus elaphus*) by the UP societies of the Caucasus in sharing aesthetic values and/or a symbolic sphere.

## Introduction

The southern Caucasus played overall a key role in human evolution, with the region of western Georgia (Imereti) being a major geographic corridor for human migrations during the Middle and Upper Paleolithic. It is also a prime location to study Neanderthal and modern human interactions during the Middle to Upper Paleolithic transition [1–5]. The study of the local Early Upper Paleolithic (EUP) and Upper Paleolithic (UP) sequences is critical to the understanding of how human populations responded to the climatic shifts during the Last Glacial [6, 7].

New research and excavations in this region since the mid-1990s show its importance as a possible route for the dispersal of AMH to northern Eurasia [1–3, 8–12]. Moreover, recent excavations with fine chronological and stratigraphic resolution indicate a discontinuous transition model between the Middle Paleolithic and the Early UP ca. 39,000–34,000 years ago with evidence of a chronological rupture between these two cultural traditions [5, 7, 13–19]. This suggests that the Greater Caucasus mountain range, which seems to have constituted a geographic-cum-cultural barrier for the migration of Neanderthals to the north, was crossed by AMH [14, 20]. Recent work also suggests that climate change around 40 Ka would have favoured the migration of AMH into this region [7, 10].

The local UP research (e.g., [13, 17–21]) had been mostly concerned with human adaptation to local circumstances reconstructed through environmental studies (e.g., palynology [22]) and reflected in hunting behaviors and the fauna prey remains [2, 23–30], as well as the developments and evolution of the chipped stone technology [13, 17]. Actual human remains are quite few [31].

Currently, the earliest Caucasian EUP dates derive mainly from sites on the northern slopes of the Greater Caucasus (Mezmaiskaya and Korotkaya) [10, 11] and those located on its southern slopes (Dzduzuana, Ortvale Klde, Bondi Cave, Aghitu-3 Cave) [3, 13, 32]. Most of those dates cluster around 39 Ka CalBP, yet more recent dating endeavours place the beginning of the UP at 46.7/43.6 Ka calBP [5]. It appears that the regional EUP assemblages from both the northern and southern Caucasus have similar techno-typological features. These EUP assemblages are rich in retouched and backed bladelets resembling more the contemporaneous Levantine Ahmarian than the European Early Aurignacian and the ‘Classic’ Levantine Aurignacian [7, 13, 14].

Personal ornaments and bone tools, engravings on objects and cave walls, and burial practices, are among the cultural features considered as reliable proxies for the emergence of symbolically mediated behaviour (SMB) [33–43]. Indeed, personal ornaments constitute a valuable archaeological category since a) they indicate shared aesthetic values and may serve as markers of social identity (be it a status [e.g., age-grade] or group [e.g. family, clan]; b) they have been used by a large number of ethnographically well-documented traditional societies combining both, their aesthetic and symbolic merits; c) they are common at UP sites, and d (they occur during this period in a great typological variety [44–58]. It is thus essential to try to assess the preferences in a regional selection of the raw materials, supports and shapes of the ornaments–if any–and compare it with other areas. Bone implements are well represented in most of the sites, with assemblages that comprise mainly simple/massive based points, hunting weapons, and bone awls, but also eye needles and personal ornaments [3, 11, 13, 18, 19, 28]. However, worked bone items and personal ornaments have contributed little to the characterization of the UP assemblages from Northern Caucasus (e.g., Mezmazkaia Cave [11]) and Southern Caucasus (e.g., Dzudzuana Cave [13]) as these studies portray mainly a typological approach, while technological and use-wear analyses are rare or missing all together.

Here we present an archaeozoological, technological and use-wear study of pendants from two UP Southern Caucasus assemblages, Dzudzuana Cave and Satsurblia Cave, located 30 km apart in the Imereti region, Georgia. The set encompasses specimens both from the EUP (Satsurblia layers B/V, B/IVb) and later UP (Dzudzuana Unit C; Satsurblia layers A/III, B/III, BIVa), from ca. 43/39 to 25.5/24.4 Ka calBP. The ornaments were made mostly on teeth by a perforation of the root to be suspended as pendants or attached to garments. Other specimens are made on bone. According to technological and experimental analyses (see below), both the manufacturing marks made during the preparation of the items and the use-wear traces indicate that the modified items served as personal ornaments.

While the chipped-stone techno-typology seems to demonstrate a local development of the region’s EUP entities, the bone tools and personal ornaments show similarities with comparable items from the European and Levantine Aurignacian and some sets from the initial UP of Central and Northern Asia. Such analogies suggest a link between the symbolic sphere of cultural entities in the Caucasus, Europe, the Levant, and other Asian regions during the early UP. Regarding the personal ornaments, the importance accorded to the red deer within the symbolic sphere of the Caucasus UP hunter-gatherers is highlighted by the selection of this taxa to fabricate almost 50% of the pendant assemblages. In comparison, this taxon comprises only 2% of the total NISP of the faunal remains in the studied layers at Dzudzuana [25], whereas at Satsurblia it includes c. 28% of the assemblage. The results of the personal ornaments studies point to the existence of anthropological and cultural ties between the Northern and Southern Caucasus UP populations, as has been previously suggested based on cross-regional sharing of lithic techno-typological characteristics [7].

## Archaeological context

### Dzudzuana cave

**Fig 1:**
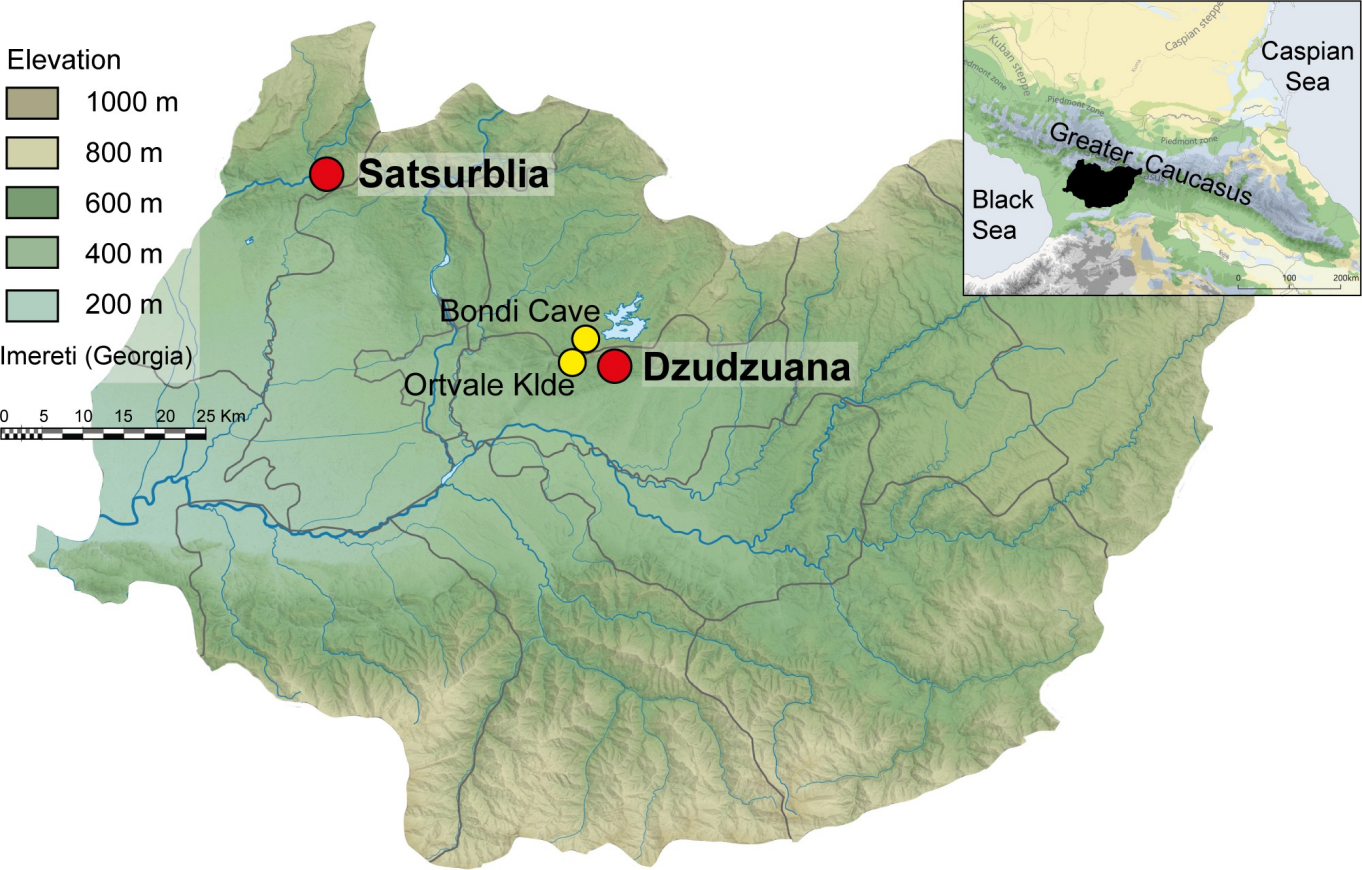
Location of Dzudzuana, Satsurblia and other Imeretian sites mentioned in the text. Main map provided by Wikimedia Commons under a Creative Commons Attribution-Share Alike 4.0 International (author Giorgi Balakhadze), original copyright 2018. Background map after Stone, T.A., and P. Schlesinger. 2003. RLC Vegetative Cover of the Former Soviet Union, 1990. ORNL DAAC, Oak Ridge, Tennessee, USA. This dataset is openly shared, without restriction, in accordance with the NASA Data and Information Policy.

Dzudzuana Cave is located in the Nekressi river valley, a tributary of the Kvirila River (Fig 1). It is situated at approximately 560 m above sea level, and 12 m above the river channel. It is a large elongated karstic cavity that emerges as a tunnel from which a small creek flows on top of the deposits [13]. The first series of excavations (1966–1975) was carried out by D. Tushabramishvili who had distinguished between two complexes, ensemble I (Eneolithic) and ensemble II (Upper Paleolithic), the latter subdivided into eight layers [59]. Faunal remains from these excavations included Caucasian tur (*Capra caucasica*) and the extinct steppe bison (*Bison priscus*), as well as red deer (*Cervus elaphus*), aurochs (*Bos primigenius*) and other mammals, in small frequencies [60].

**Fig 2:**
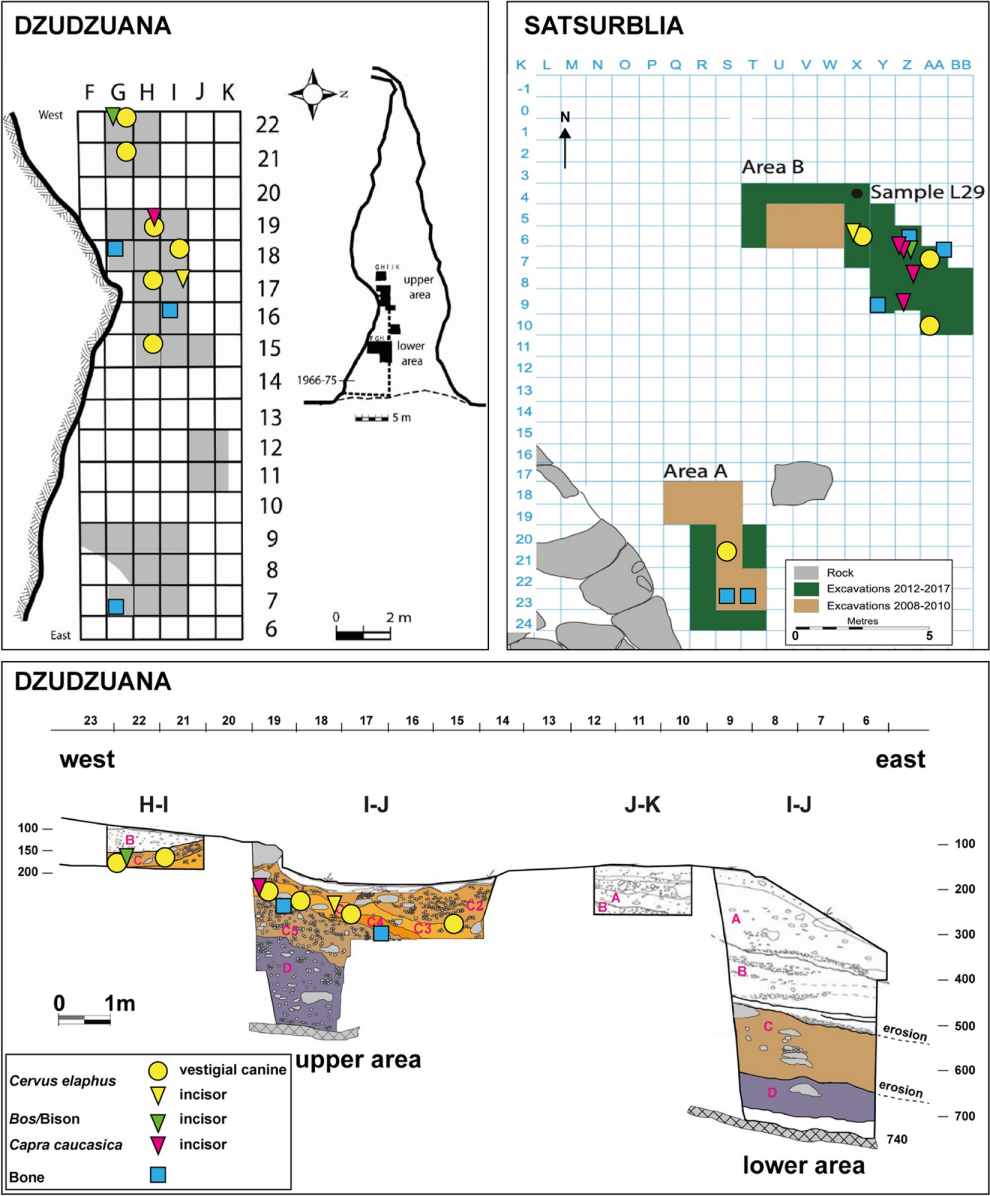
Spatial distribution and stratigraphic location of pendants from Dzudzuana and Satsurblia. On the left, spatial distribution (top) and stratigraphic location (bottom) of pendants from Dzudzuana Cave (red deer canines = yellow circles; red deer incisor = yellow triangle; caprid incisor = magenta triangle; bone pendants = blue square) plotted over the West–East sections. On the right, the Satsurblia Cave pendants spatial distribution.

A second series of excavations (1996–2008) led by T. Meshveliani and O. Bar-Yosef, were carried out at the frontal (lower area) and inner (upper area) parts of the cave, totaling ca. 16m^2^ [61] (Fig 2). Rich lithic and faunal remains were systematically collected in small excavation units of 0.5 x 0.5 x 0.05 m. All excavated sediments were wet-sieved through 2 mm mesh and the dried sediments were hand-picked for small finds. All the fauna was retained and processed according to spatial and stratigraphic locations. The total depth of the UP deposits is about 3.5 m, divided into five major stratigraphic units: layers B and C in the upper area, and layers B, C and D in the lower area. Technological and typological studies of the lithics have been summarized and reported alongside a series of radiocarbon dates obtained from bones and charcoal samples [8, 13]. The dates of the Units are: Unit D—34.5–32.2 Ka cal BP; Unit C—27–24 Ka cal BP; Unit B—16.5–13.2 Ka calBP.

The cultural material remains comprised chipped stone assemblages, bone and teeth artefacts, faunal remains, etc. Most of the chipped-stone assemblages were manufactured from a local chert variety (radiolarite) which is easily obtained either from the plateau above the cave or down in the river bed, mostly of mediocre quality. There is a very limited use of obsidian, which was brought from ca. 80–100km away [20]. The preservation of the lithics is good and the artefacts are predominantly in mint condition. The ratios of debitage items- per- tools in all units indicate that the latter were probably brought over to the cave as finished products.

The lithic assemblage of Unit C at Dzudzuana [13] is quite rich, comprises ca. 40,000 Debris items, ca. 15,000 Debitage items, ca. 725 Cores, and 2250 tools. The blade/bladelet component derives from narrow carinated cores and among the tools the dominant category is that of retouched bladelets (37.1%). The second largest category, typical for the Upper Paleolithic in general, are the endscrapers (ca. 23%), varying in type and form, on flakes or blades. Next are the burins (8.4%). The rest of the tool categories (awls and borers, notches and denticulates) comprise between 3%-to-1% of the tools. Of interest to note the presence of the Gravette and micro- Gravette points though they represent but 1% of the tool inventory. Most of the chipped stone assemblage is made on flint and chert while obsidian items comprise ca. 4% of the total.

A unique discovery are wild flax fibers (spun and dyed) recovered from the pollen samples taken on site [62]. Fibers were recovered from all units, the richest being Unit C. The micro-remains of fur, skin beetles and moth can be interpreted as evidence for working hide and flax. The samples with the highest content of flax also contained spores of the *fungus Chaetomium*, which usually grows on clothes and textiles, destroying them.

Bison (*Bison priscus*), aurochs (*Bos primigenius*) and Caucasian tur (goat) (*Capra caucasica*) are the most common taxa in all occupation levels [25: tab. 2]. Other ungulate species are represented in small frequencies and include primarily red deer (*Cervus elaphus*). It appears that the earliest occupation at the site (Unit D) contains higher proportions of Caucasian tur, while Unit C contains higher proportions of steppe bison and aurochs. In Unit B the percentages of all three are quite similar. It could be that the differences in species abundance between the units reflect differences in the season of occupation. The high frequency of the Caucasian tur in Unit D may indicate hunting activities in late autumn or winter when the herds descended into the higher part of the forests. The high frequency of steppe bison in Unit C may result from hunting in early spring or summer when bison herds climbed to the woodland in the mountainous areas (see [63, 64] for detailed accounts of the behavioural ecology and seasonal migration of Caucasian tur and steppe bison).

The taphonomic history of Dzudzuana Cave shows that the bone assemblage of each unit was accumulated by the same subsistence strategy. This is indicated by the similarity of species composition and the demographic profile, as well as the similarity of carcass processing and marrow extraction techniques.

**Fig 3:**
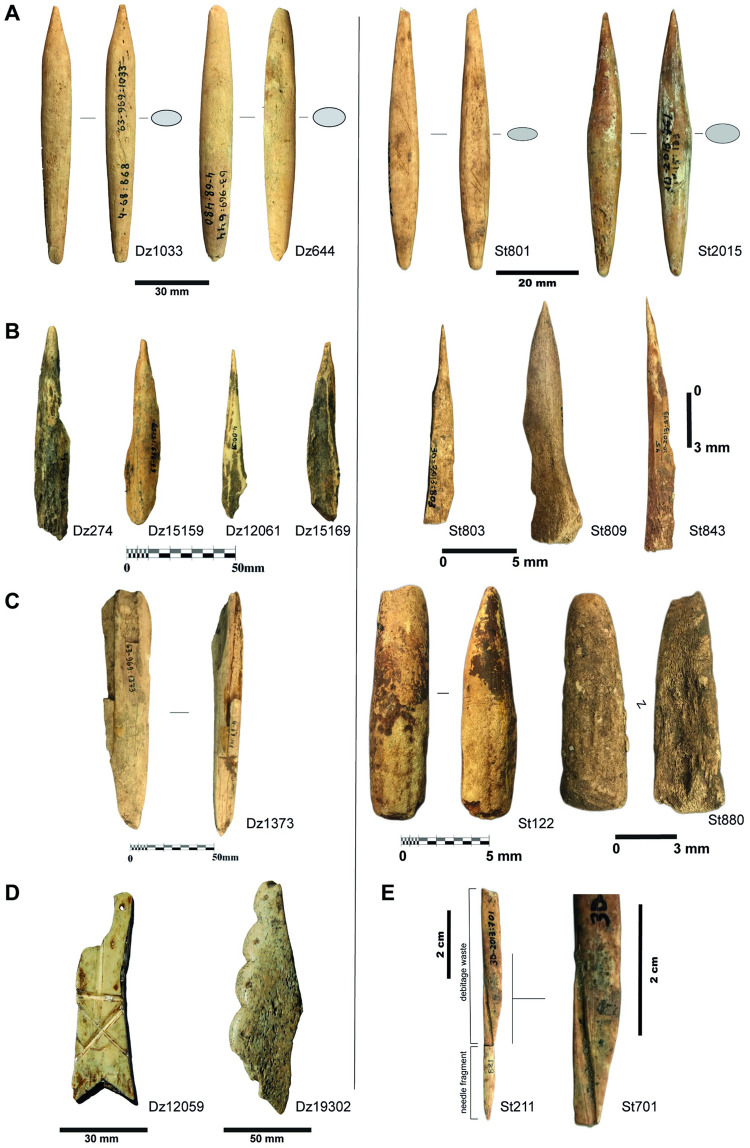
Bone tools examples from Dzudzuana (left) and Satsurblia (right). Projectile points (A); awls (B); ‘intermediate pieces’ (chisels) (C); notched and incised bone fragments from Dzudzuana (D); bone needle from Satsurblia and debitage waste linked with needle production (E). Specimens housed at the prehistory storage facilities of the Georgian State Museum (Tbilisi, Georgia). Photos by J.-M. Tejero.

The bone tools assemblage of the UP units in Dzudzuana constitute a rich sample with ca. 250 items including tools, personal ornaments, notched or incised bones (‘decorated’), and “technical pieces” (waste, blanks, preforms). Projectile points–hunting weapons–and awls are the best-represented categories (Fig 3). There are also a few specimens of other tool-types, such as ‘intermediate pieces’ probably used in indirect percussion (namely as chisels), as has been demonstrated through both technological and experimental studies [65, 66].

Whenever the base of the projectile points is preserved, it is a simple/massive point, of the variety defined as ‘elongated objects with a pointed distal tip, a variable cross-section (mostly elliptical) and a simple hafting system’ [67:1, 68]. Though such items are frequently fabricated on antler, especially in the earlies phases of the UP both in Europe and the Levant [69–77], in Dzudzuana they are mostly of bone. Indeed, antler working is quite rare at the site. Only two projectile points seem to have been made on antler *contra* the findings from Satsurblia (and see below).

The preservation of the items is quite good. Although around half of the assemblage display eroded surfaces and sediment concretions, most of these taphonomic alterations do not prevent the technical assessment of the work marks. Functional breakages, identified based on experimental and technological literature [78–81], are observed on ca. 35% of the bone projectile points and awls.

### Satsurblia cave

Satsurblia cave is located in the Sataplia-Tskaltubo karst massif in the same region as Dzudzuana (Fig 1). It is situated at 360 m above sea level. The cave is around 125 meters long. The site was discovered in 1975 by AI. Kalandadze [82], who subsequently excavated it sporadically in 1976, 1985–88. Excavations were carried out also by K. Kalandadze in 1989–1992 [83], and by T. Meshveliani in 2008–2010. Later excavation campaigns at Satsurblia were conducted between 2011–2017 led by T. Meshveliani and R. Pinhasi and an international team of experts in the framework of a multidisciplinary project on the UP in the region [17].

The later excavations were conducted in two areas, Area A in the north-western part of the cave, near the entrance, and Area B in the rear of the cave, adjacent to a trench previously excavated by K. Kalandadze (Fig 2). Both areas revealed stratigraphic sequences comprising Pleistocene (Upper Palaeolithic) and Holocene (Eneolithic and more recent) deposits. The exposed stratigraphic sequence of Area A corresponds to four main archaeological strata (labelled A/I, A/IIa, A/IIb, A/III) A/IIa-A/IIb dating to 17.9–16.2 Ka calBP and A/III to 24.4–25.5 Ka calBP. Area B comprises 6 archaeological layers (B/I, B/II, B/III, B/IVa, B/Vb, B/V) with a calibrated age of 24.4–25.5 Ka calBP for layers B/III-B/IVa and 31.5–32.2 ka calBP for layers B/IVb-B/V.

The lithic assemblages from the relevant layers in Satsurblia are as yet not fully published (but see [17]). They also vary in sizes (e.g., A/III comprises 232 tools, 56 cores, ca. 3200 Debitage and ca. 2800 Debris; B/III– 742 tools, 192 cores, ca.12,000 Debitage and ca. 12, 000 Debris; B/IVa– 750 tools, 186 cores, ca. 8800 Debitage and ca. 10,000 Debris; B/IVb– 762 tools, 171 cores, ca. 8800 Debitage and ca. 7500 Debris. B/V [still under excavation]– 100 tools, 30 cores, ca. 1200 Debitage and ca. 600 Debris). Still, their study revealed that thought their time-range is quite extensive their basic characteristics are quite similar. Thus, the dominant tool category are the retouched bladelets (from 25% in B/V to 20% in A/III), and one can add here the backed bladelets (17% to 14%) which makes the bladelets the major component of the tool categories. The main difference between the assemblages of the EUP and those of the later UP is in the reduction of the former (retouched bladelets) and the rise in the latter (backed bladelets). The next category is that of the endscrapers (20%-15%), always outnumbering the burins (ca. 12% on the average, with one exception–B/III). Just as in Dzudzuana the rest of the categories, typical of the UP are represented by single percentages. The Gravette and micro-Gravette points are present in 1.3% to 1%. Also, here the carinated cores are present in significant numbers (between 13% to 20%.

The faunal analysis indicates that the subsistence focused on hunting of wild boar (*Sus scrofa*), and red deer (*Cervus elaphus*) as well as Caucasian tur (*Capra caucasica*) and wild bovines (*Bos*/Bison). The Satsurblia UP faunal assemblages differ from those reported from other UP sites in the region in which hunting focused on *bos*/bison or wild goat [17].

The bone assemblage exhibits excellent preservation as evidenced by the presence of a whole range of bone densities, including porous parts such as sternum fragments. Bone preservation does not seem to vary among taxa. The long bones show minor signs of surface weathering, indicating rapid burial of finds and the cave’s protective conditions. Traces of carnivore bone ravaging activities are few, observed on the remains of all ungulate taxa. Rodent gnaw marks are also present in low numbers. It appears that scavenging animals had only secondary access to the food remains [17].

Preliminary analysis of breakage patterns and bone surface modification reveals that the dominant agents of bone accumulation and bone damage were the humans. Virtually all ungulate long bones were split open to obtain marrow, evident by the high ratio of fresh (green) fractures (over 80% and following the typology of Villa and Mahieu [84]). Butchery marks are observed on boar and cervid bones, representing all butchery and carcass processing stages (skinning, dismemberment, and filleting).

Technical features of the production processes of bone tools are identical in both sites (Fig 3). The bone awls were modified using a single, simple technique—scraping. On the other hand, the hunting weapons were produced using a combination of techniques, following a process defined by Averbouh [85], of a complex operational sequence.

The technical features of the production processes of osseous raw material should yet to be assessed. Indeed, a renewed technological study of the bone tool production technology from several Southern Caucasus assemblages is currently underway.

## Materials and methods

The herein discussed assemblages of personal ornaments recovered from Dzudzuana and Satsurblia caves consist of a total of nineteen perforated or grooved teeth, eight perforated or grooved bones and one possibly antler fragment, also with a perforation (Table 1). We have not included in the study ‘decorated (drilled, incised, notched) bones, shells and pendants made on stone from Dzudzuana described in previous publications [13]. All the pendants from Dzudzuana were found in Unit C, one in sub-layer 3 and one in sub- layer 4 (Fig 2). The items from Satsurblia derive from area A (N = 3) and area B (N = 13) of the recent excavations. All pendants from area A have been recovered in layer A/III. Two pendants from area B were found in layer B/V, three in layer B/IVb, one in layer B/IVa, and seven in layer B/III. No permits were required for the described study, which complied with all relevant regulations.

**Table 1:** Pendants from Dzudzuana and Satsurblia caves.

Site	Number	Area	Unit/Layer	Chrono-cultural attribution		Species	Raw Mat.	Anatomical support	Sex	Side	Observ.
Dzudzuana	19358	—	C	UP	H15b	*Cervus elaphus*	tooth	vestigial canine	male	right	
Dzudzuana	15161	—	C	UP	G23d	*Cervus elaphus*	tooth	vestigial canine	male	left	
Dzudzuana	12077	—	C	UP	H19b+d	*Cervus elaphus*	tooth	vestigial canine	male	right	
Dzudzuana	19308	—	C	UP	I18b	*Cervus elaphus*	tooh	vestigial canine	female	right	
Dzudzuana	19371	—	C	UP	H17b	*Cervus elaphus*	tooth	vestigial canine	female	right	
Dzudzuana	19344	—	C	UP	G21b	*Cervus elaphus*	tooth	vestigial canine	female	left	
Dzudzuana	15080	—	C	UP	G23c	*Bos/*Bison	tooth	incisor	—	left	
Dzudzuana	15128	—	C/layer 3	UP	I17b	*Cervus elaphus*	tooth	incisor	—	indet.	red deer vestigial canine like-shape
Dzudzuana	12056	—	C	UP	H19b+d	*Capra caucasica*	tooth	incisor I1/2	—	left	
Dzudzuana	19287	—	C	UP	G18a	indet	bone	—	—	—	red deer vestigial canine like-shape
Dzudzuana	15153	—	C/layer 4	UP	I16a	indet	bone	indet	—	—	red deer vestigial canine like-shape
Dzudzuana	12066	—	C	UP	G7c	indet	bone	indet	—	—	red deer vestigial canine like-shape
Satsurblia	St6	B	V	EUP	AA10c	*Cervus elaphus*	tooth	vestigial canine	male	—	
Satsurblia	St7	B	V	EUP	Z8b	*Capra* sp	tooth	incisor	indet.	—	
Satsurblia	St2	B	III	UP	Y6d	*Cervus elaphus*	tooth	vestigial canine	male	—	
Satsurblia	St3	B	III	UP	AA7c	*Cervus elaphus*	tooth	vestigial canine	female	—	decorated (notched)
Satsurblia	St8	A	III	UP	S21	*Cervus elaphus*	tooth	vestigial canine	male	—	
Satsurblia	St9	B	IVb	UP	Z7b	*Bos/*Bison	tooth	incisor	indet.	—	
Satsurblia	St4	B	III	UP	Y6d	*Cervus elaphus*	tooth	incisor I1	indet.	—	decorated (notched)
Satsurblia	St11	B	IV	UP	Z7d	*Capra* sp	tooth	incisor	indet.	—	
Satsurblia	St15	A	III	UP	S23	*Cervus elaphus*?	antler	indet.	—	—	
Satsurblia	St16	B	III	UP	Y6b	*Capra* sp	tooth	incisor	indet.	—	
Satsurblia	St1	B	IV	UP	Y8c	indet.	bone	indet.	—	—	
Satsurblia	St5	B	III	UP	AA7a+b	indet.	bone	indet	—	—	
Satsurblia	St10	B	IVb	UP	Z9c	*Capra* sp.	tooth	incisor	—	—	
Satsurblia	St14	A	III	UP	T23d+c	indet.	bone	indet.	—	—	
Satsurblia	St17	B	III	UP	Y6b	indet.	bone	indet	—	—	
Satsurblia	St21	B	III	UP	BB8a	Indet.	bone	indet.	—	—	

The studied material is housed at the prehistory storage facilities of the Georgian State Museum (Tbilisi, Georgia) (Table 1). The taxonomic identification of the items is based on the comparative osteological collections of the National Natural History Collections, The Hebrew University of Jerusalem and the University of Haifa through prior research [52]. Published criteria were also consulted for taxonomic identification, e.g., Brown and Chapman [86] for red deer.

The distinct sexual dimorphism of red deer [87, 88], is also expressed in the shape and size of their canines [89]. Over the evolution of the family *Cervidae*, hornless ruminants predated the antlered ones. The size of the tusk-like upper canine teeth has tended to be inversely correlated with the size of the antlers, as if ‘holding on’ to the former compensated for the lack/small size of the latter. As the antlers became longer, the canines became shorter, eventually disappearing. While in most cervids they have been lost some species like red deer (*Cervus elaphus*) have retained vestigial canines [87].

As changes occur through life in the shape, root and size of the canines, the specimens were examined for age and sex, following the methods of d’Errico and Vanhaeren [90]. Apparently, male vestigial canines are broader than those of the females and with wear, the fully-grown shape in young males becomes triangular. Conversely, crowns of young females are pointed, and those of older specimens are rectangular. The roots of vestigial male canines are square or trapezoid while those of females are rectangular or V-shaped.

We recorded morphometric variables for each canine. These include occlusal wear stages, stages of root development, state of closure of the pulp cavity, and wear removal of the disto-lingual-cervical lobe. Metric variables include crown width, length and thickness, width and length of the occlusal wear facet, maximum width, apex width, root thickness and root length (Table 2).

**Table 2:** Red deer (*Cervus elaphus*) vestigial canines pendants morphometry (see d’Errico and Vanhaeren 2002).

Number	Root length	Root width	Root apex width	Root thickness	Ratio L/T	Crown L	Crown W	Crown T	Length of occlusal surface	Width of occlusal surface	Wear stage of occlusal surface	Root apex	Pulp cavity	Distolinguocervical lobe
19358	14	10	7	4	2.5	16	14	8	11	6	affected	closed	not visible	present
15161	16	14	9	4	3.5	12	14	9	22	8	highly	closed	not visible	absent
12077	18	14	11	4	4.5	13	13	11	22	12	highly	closed	not visible	absent
19308	14	8	5	4	1.7	8	11	7	11	7	highly	closed	not visible	absent
19371	12	9	6	5	1.3	7	8	7	11	8	highly	—	—	absent
19344	12	9	6	5	1.3	12	12	7	11	6	affected	—	visible	present
St2	17	15	8	5	3.4	12	15	9	19	8	highly	closed	not visible	absent
St3	12	9	6	5	2.4	16	12	9	10	6	affected	closed	not visIble	present
St6	20	16	11	6	3.3	15	17	11	11	8	affected	—	—	present
St8	15	12	9	5	3.0	12	14	9	14	7	affected	closed	not visible	present

Abbreviations: L = length; W = width; T = thickness.

For the description of the specimens’ modification, we followed the methodology of Barge-Mahieu et al. [91], White [44, 45, 92], Vanhaeren [93] and d’Errico and Rigaud [94], among others. We described the perforation process, dimensions, location, preparation modes and perforation techniques (e.g., diameter, shape, the distance between the tooth buccal and lingual edges, distance between the perforation and the root base).

Technological and use-wear analyses were conducted using both a stereomicroscope Leica S8APO with led light LZ, and an Olympus SZX16 with KZ1600 LED source (magnification: 10–115x). Stereomicroscope images were taken with an Olympus SC50 camera coupled to the microscope and recorded with Olympus CellSens software.

## Results

### Taxonomical and sexual identification, preservation

**Fig 4:**
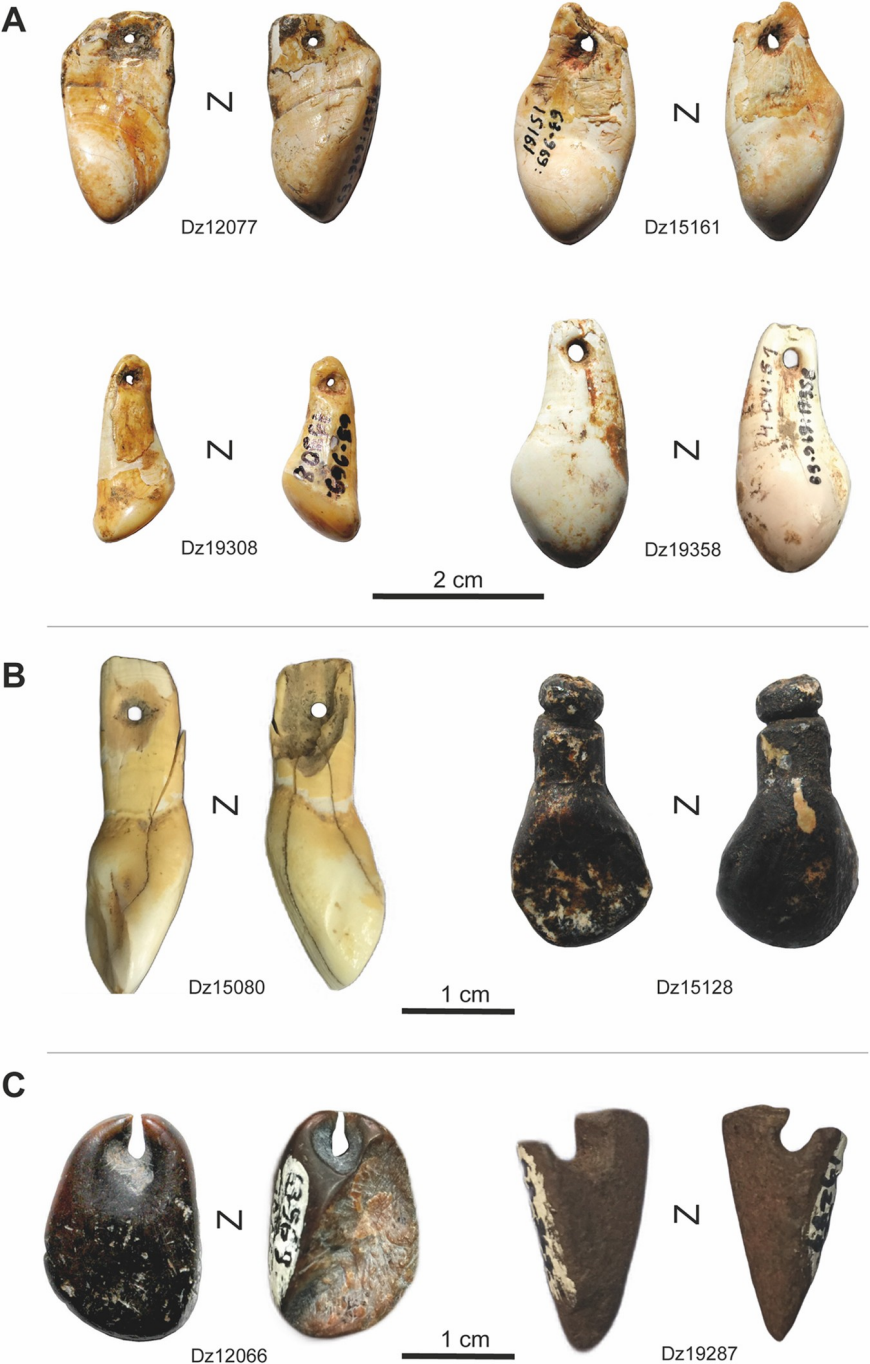
Pendants from Dzudzuana cave. Red deer vestigial canines (A); ungulate incisors (B); bone pendants (C). The specimens are housed at the prehistory storage facilities of the Georgian State Museum (Tbilisi, Georgia). Specimens housed at the prehistory storage facilities of the Georgian State Museum (Tbilisi, Georgia). Photos by J.-M. Tejero.

**Fig 5:**
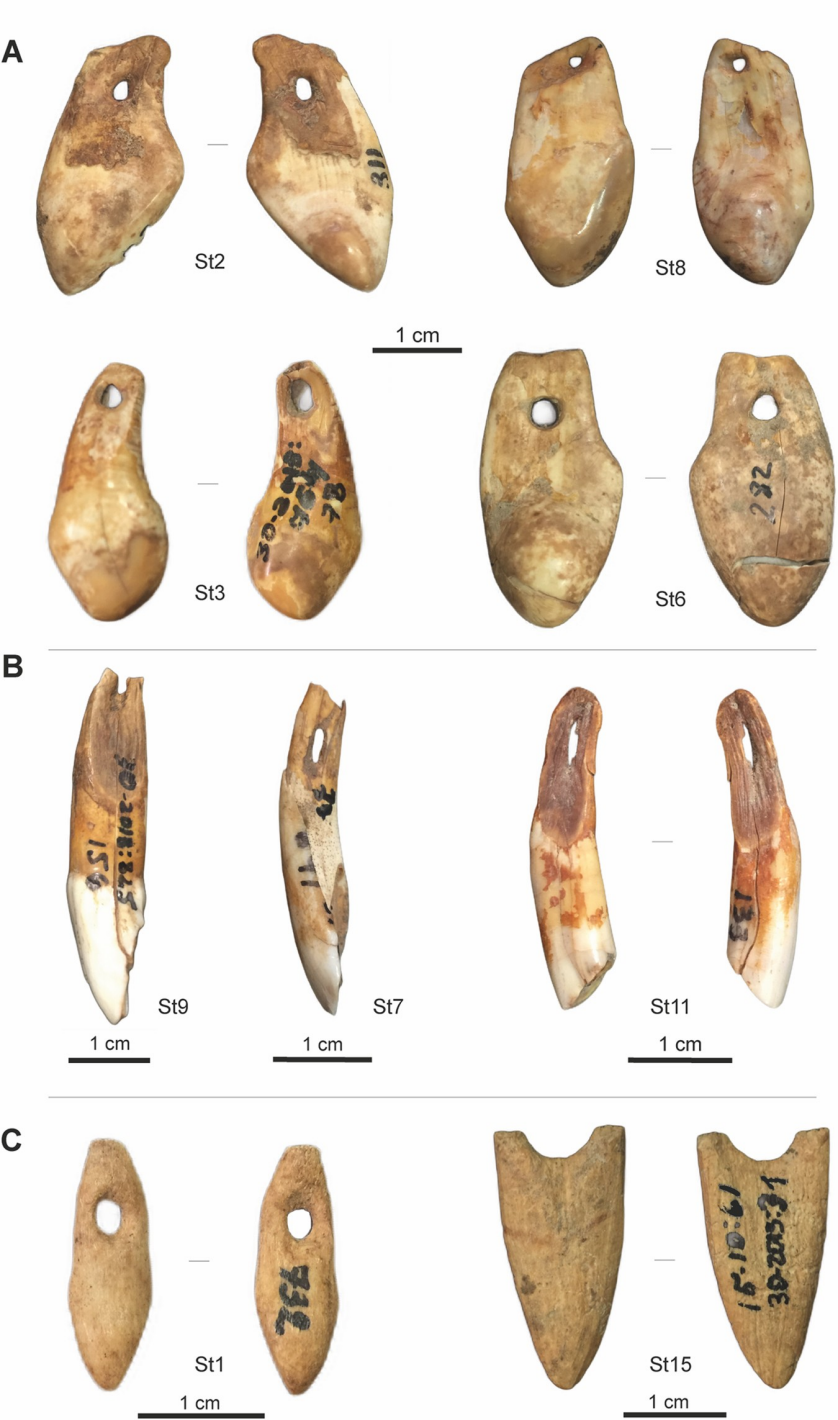
Pendants from Satsurblia cave. Red deer vestigial canines (A); ungulate incisors (B); bone pendants (C). Photos by J.-M. Tejero.

All the data obtained through the current study is presented in Tables 1–4. Of the teeth pendants, the majority–ten specimens—are vestigial canines of red deer (four right, two left and four indeterminate), and based on the size ratio six of them are of males and four are of females. The other nine teeth pendants represent two red deer incisors, two *Bos*/Bison incisors and five incisors of caprinae (*Capra caucasica/Capra* sp.) (Figs 4 and 5, Table 1).

**Table 3:** Detailed description of the pendants attaching system.

Number	Type	Preparation of the surface	Perforation/grooving procedure	Perforation shape	Diameter (mm)	Distance between rigth and left edges (mm)	Distance between the perforation and the pendant base (mm)
Dz19358	perforation	—	bifacial gouging + rotational scraping	circular	3 × 3	3/4	3
Dz15161	perforation	—	bifacial gouging	elliptical	4 × 3	3.4/4.5	—
Dz12077	perforation	—	bifacial rotational scraping	circular	3 × 3	3/4	3
Dz19308	perforation	—	bifacial gouging + rotational scraping	circular	3 × 3	2/2	3
Dz19371	perforation	abrasion	bifacial gouging	—	—	2/2	—
Dz19344	perforation	abrasion	bifacial rotational scraping	circular	—	2/2	—
Dz15080	perforation	scraping	bifacial gouging	circular	3.5 × 3.5	3/3	4
Dz15128	groove	root transversal sectioning	periferical gouging	—	—	—	—
Dz12056	perforation	scraping	bifacial gouging	—	—	—	—
Dz19287	perforation	—	bifacial rotational scraping	circular	3 × 3	2/4	—
Dz15153	indet. (unfinished?)	—	—	—	—	—	—
Dz12066	perforation	—	—	—	—	—	—
St1	perforation	scraping	bifacial rotational scraping	circular	3 × 3	2/3	5
St2	perforation	scraping	bifacial gouging	subcircular	5 × 4	3/3	6
St3	perforation	abrasion	bifacial gouging + rotational scraping	circular	4 × 4	3/3	4
St4	perforation	—	bifacial gouging	elliptical	5 × 3	8/9	—
St5	perforation	—	bifacial gouging + rotational scraping	subcircular	3 × 2.5	3/4.5	3
St6	perforation	—	bifacial rotational scraping	circular	5 × 5	4/4	6
St7	perforation	scraping	bifacial gouging	elliptical	5 × 3	—	—
St8	perforation	—	indirect percusion	trapezoidal	4 × 3	3/4	—
St9	perforation	scraping	bifacial gouging	elliptical	4 × 2	3/3	—
St10	perforation	—	bifacial gouging	elliptical	4 × 3	2/2.5	5
St13	perforation	scraping	bifacial gouging	elliptical	5 × 3	3/3	8
St14	perforation	—	—	—	—	—	—
St15	perforation	—	bifacial gouging	—	—	—	—
St16	perforation	—	bifacial gouging + rotational scraping	subcircular	3.5 × 3	2/2	4
St17	perforation	—	bifacial gouging?	circular	3 × 3	3/3.5	3
St21	perforation	—	bifacial gouging + rotational scraping	subcircular	4 × 3.5	4/3	4

**Table 4:** Tooth removal marks, use-wear marks, functional breakages and ocher stains.

Number	tooth removal marks	Use wear marks	Use-wear marks location	Functional breakage	Ochre stains	Ochre stains location
Dz19358	yes	polish	Upper (distal) part of perforation	no	yes	hole/crown and root bucal and vestibular faces
Dz15161	yes	polish	Upper (distal) part of perforation	no	yes	inside and outside hole on bucal and vestibular faces
Dz12077	—	—	—	no	no	—
Dz19308	—	polish	Upper (distal) part of perforation	no	yes	inside hole
Dz19371	—	—	—	yes	yes	inside hole
Dz19344	—	—	—	yes	yes	inside/outside hole
Dz15080	—	polish	Upper (distal) part of perforation	no	yes	inside/outside hole on bucal and vestibular faces
Dz15128	—	polish	Upper (distal) part of perforation	no	no	—
Dz12056	—	—	—	no	no	—
Dz19287	—	—	—	yes	yes	inside hole
Dz15153	—	—	—	no	no	—
Dz12066	—	—	—	yes	yes	inside/outside hole
St1	—	—	—	no	no	—
St2	yes	polish	Upper (distal) part of perforation	no	yes	inside hole
St3	yes	polish	Upper (distal) part of perforation	no	yes	inside/outside hole on bucal and vestibular faces
St4	—	polish	Upper (distal) part of perforation	no	no	—
St5	—	polish	Upper (distal) part of perforation	no	no	—
St6	—	polish	Upper (distal) part of perforation	no	no	—
St7	—	—	—	yes	no	—
St8	—	polish	Upper (distal) part of perforation	no	no	—
St9	—	—	—	yes	no	—
St10	—	—	—	no	yes	inside hole
St13	—	—	—	no	yes	inside hole
St14	—	—	—	yes	no	—
St15	—	—	—	yes	no	—
St16	—	—	—	no	no	—
St17	—	polish	Upper (distal) part of perforation	no	no	—
St21	—	—	—	no	yes	inside hole

While most of the teeth pendants could be identified to the type, species, sex and side (see Table 1), the taxonomical and anatomical origin of the perforated or grooved bones cannot be determined because of the pendants’ small size and the extensive modification of the bone surface to manufacture the pendant. Based on the ratio between the–thin–cortical bone tissue and the trabecular osseous tissue, and the alveolus’ morphometrics, one pendant (Dz12059) could have been made on a mammal rib fragment. Another item (St15) is perhaps made of antler, based on the presence of antler-like trabeculae in its lower face.

No particular spatial association was observed between the pendants and other non-lithic artefacts (bone tools, incised bones) or structures (fireplaces) in Dzudzuana. Thus, of the twelve pendants from this site only two–a red deer vestigial canine and a goat incisor–were found in the same square (H19b+d). In the case of Satsurblia, four of the sixteen pendants derive from the same square (Y6) in Area B, layer B/III (a red deer vestigial canine and an incisor, one Caucasian tur incisor and a perforated bone) (Fig 2). One of these specimens displays marks of having been subjected to a combustion process. Its surface was burned, showing a homogeneous colour and patina (soft brown) usually associated with low temperatures and short time exposition to a combustion process [95]. Since no other piece was burnt it is not possible to speculate about the purpose (if any) of the thermic process. Alternatively, it could be merely incidental (e.g., a piece discarded into a fire or close to it), especially since the burned surface of the tooth seems to be restricted to the vestibular face [96, 97].

Most of the pendants are well preserved. Some display sediment concretion, exfoliations (most of the enamel in the lingual face), manganese spots (one specimen from Dzudzuana) and desiccation fissures. Except for two specimens from Dzudzuana and two from Satsurblia, all others are complete or almost complete (small fragment losses in some cases). Besides the above-described item from Satsurblia, two other teeth pendants and one bone pendant from Dzudzuana are burned, with nonhomogeneous coloured surface varying from dark brown to black. The uneven distribution of the burned colour and the black tonality showing a high and likely uncontrolled exposure of the items to fire action [97, 98] suggest that it was an incidental rather than a deliberated thermic process.

**Fig 6:**
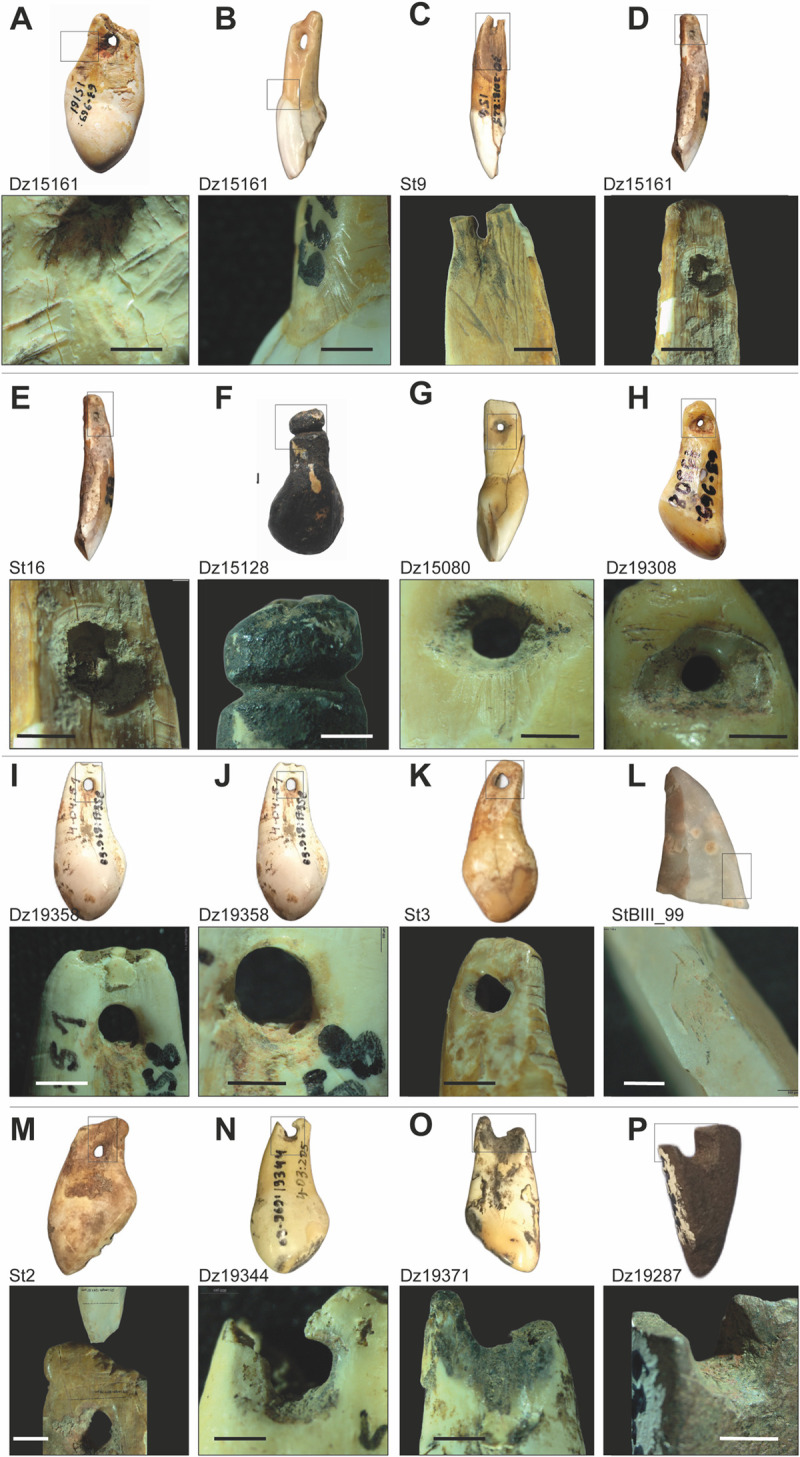
Detailed stereo-microscopy photographs of pendants. Oblique incisions likely produced to remove the teeth from the alveolar cavity (A, B); Longitudinal scraping on the surface of the roots before perforation (C, D); circular marks on the hole resulting from a rotating action to perforate the teeth (E); transversal segmentation of the root by sawing and peripheral gouging by scraping (F); Longitudinal incisions marks resulting from the perforation by scraping (G); circular perforations modified by use-wear resulting in a polish lobe of the hole (G–K); flint burin from layer B/III of Satsurblia bearing ochre remains and compatibility between its distal (active) part and the scraped and perforated surface of a teeth from the same layer (L, M); ochre stains on the holes (H, J, P); red deer vestigial canine from Satsurblia decorated with transversal notches on its root (K); functional breakages of the perforations (N–P). All scales = 1 mm. Magnifications 7-16x. Photos by J.-M. Tejero.

The extent to which the worked teeth were derived from hunted animals or were rather recovered through scavenging remains unknown. Nevertheless, as noted by several authors, vestigial deer canines disperse quickly after death as they detach easily from the maxilla [94]. This makes it more likely that the perforated teeth originate from hunted animals. Indeed, four of the teeth display incisions on the vestibular face in the junction between the crown and the root (the base of the disto-lingual-cervical lobe). The incisions are short and thin, oblique, and perpendicular, relative to the main axis of the piece (Fig 6A and 6B). We interpret them as cut-marks to extract the teeth from the alveolar cavity, which reinforces the assumption that teeth were removed from a fresh carcass.

The wear signs of Palaeolithic personal ornaments indicate long- term use (see below), making it challenging to determine whether they were produced on-site or brought in as finished products from outside. A high degree of mobility is assumed for Palaeolithic personal ornaments tied in with their owners’ mobility [99]. Assessing their lifespan and tracking their movement through a given territory is indeed a rather speculative exercise. However, the *in situ* working of animal raw material (bone and antler) is demonstrated by the presence, at both Imereti sites, of blanks and waste associated with the production of projectile points, awls, chisels and needles (Fig 3). This suggests that at least some of the teeth and bone ornaments could have been produced on site. Indeed, one of the specimens analysed may be an unfinished–preform–pendant. It is a bone fragment from Dzudzuana (Dz15153) with a red deer vestigial canine like-shape but its suspension system is yet not implemented.

### Description of pendants and production techniques

All perforations or grooves are located along the root of the tooth or in the proximal (basal) part in the bone pendants. In several tooth pendants the root surfaces were scraped (a canine of red deer, two incisors of big bovid, and four caprine incisors) or abraded (three vestigial canines) prior to perforation. The manufacturing sequence is evidenced by the overlapping of the perforation marks over the scraping and abrasion ones (Fig 6C and 6D). Preparation-scraping or abrasion might have been done to clean the root and smooth the surface at the hole’s starting point. The preparation surface is restricted in all the teeth pendants to a limited extent of the root (between 8-12mm) in the area where the perforation is located.

The attachment or suspension system of the pendants was made by two distinct techniques–bifacial gouging or bifacial rotation–in sixteen cases (Fig 6E). A single specimen was perforated by indirect percussion. A combination of the two techniques consisting of bifacial gouging to start the hole then finishing by a bifacial rotation is observed in six pendants. The attachment system implemented by a groove was performed by a controlled peripheral gouging action (“*rainurage*”) as described for some European Aurignacian ornaments [45, 100] (Fig 6F).

While the natural original shape was kept in many of the teeth except for one incisor from Dzudzuana, the bone fragments were intensely worked to attain the desired form–mostly oval and similar in size and shape to that of the red deer vestigial canines. Thus, besides the perforation made by bifacial gouging and rotation, the bones were also intensely scraped. It seems that additionally abrasion or polish were applied to finalize the shaping of the bone pendants as evidenced by the marks covering the whole surface of the items, clearly related to the manufacturing process rather than a potential use-wear (see below).

No particular technical behaviour vis-à-vis the manufacture of the pendants at each site is observed. The same is true regarding the raw material chosen, whether teeth or bone. Indeed, one can consider the technical diversity in the pendants’ manufacture as similar to that discerned in the European Aurignacian [45], the Levantine Aurignacian [52], and the IUP from Central Asia [101] which portray flexibility in the techniques employed in ornament production.

Regarding the perforated items, most of the perforations have circular or sub-circular shape. In contrast, four specimens have elliptical holes and one item—a trapezoidal perforation (Fig 6G–6J, Table 2). These perforation forms are determined by the techniques employed. Nevertheless, some of the circular holes seem to be slightly modified later, most likely through use (see below), resulting in a subcircular form (Fig 6G–6L). The perforation cross-section is conical in almost all the items since they were perforated from both faces. Perforation diameter values are quite regular in all specimens, varying between 5 and 3 mm (Table 3). Holes are located at the root of the teeth centred from the edges and the root apex. Bone pendants holes are situated at the proximal part of the bone fragments. The distance from the lateral edge is also regular in all pendants. The hole is at the same range of length from both edges (between 2 and 4 mm), and in the teeth is equidistant between the end of the root and the beginning of the crown (Table 3). This location was likely chosen to avoid accidents during the perforation process and ensure the solidity of the teeth or bone fragment against traction forces, whichever way they were used (as beads in a necklace, bracelet, attached to clothes, or otherwise).

Two red deer teeth pendants from Satsurblia were decorated with deep (a vestigial canine) or more superficial (an incisor) notches made by accurate sawing (Fig 6L). Notched ‘decorations’ are also observed on some bone fragments from Dzudzuana and Mezmaiskaya published previously [7, 11, 13]. Such decorative motif constitutes a recurrent pattern within contexts associated with AMH from the African Middle Stone Age (MSA) up to the Eurasian UP [34, 38, 51, 101–104], while a single specimen of a notched bone is associated with the Micoquian techno-complex considered to be produced by Neanderthals [105].

Particularly interesting is the Dzudzuana’s incisor likely modified to look like a vestigial canine of a red deer (Figs 4B left, 6F). A peripheral sawing and then a bending action removed the distal part of the root. The groove was accomplished by sawing, with visible start and finish points. Its morphometrics (length and width) match well the vestigial canine ones, clearly differing from those of unmodified incisors. While the shape of deer canine pendants was at times imitated in various raw materials (bone, antler, stone) by Palaeolithic hunter-gatherer groups in Eurasia, including the Caucasian region [7, 11, 46, 106, 107], as observed also in Dzudzuana and Satsurblia, it was nevertheless rarely done by modifying other teeth.

Twelve of the items, from both sites, bear ochre stains inside and outside of the perforation on the buccal and vestibular faces of the tooth (Fig 6I, 6J and 6P, Table 4). Presence of ochre *per se* among the archaeological remains as well as the presence of modified ochre fragments have been considered as an indication of symbolic behaviour during the African MSA [108–113] and the European and Levantine Middle Palaeolithic [38, 107–109]. Exponential growth in scope and quantity is observed from the beginning of the UP onward [34, 38, 101]. In the current study, the presence of ochre exclusively on the perforated pendants but not on the grooved items, can indicate that it was used for its abrasive properties to facilitate hole perforation. Indeed, combined with a small amount of water, ochre is very useful in increasing the penetration power of the lithic tool used to make the hole [52]. A burin found in layer B/III from Satsurblia exhibiting ochre stains on one of its edges could have been used to perform the pendants holes. The width of the burin distal part and the scraped and perforated surface of one of the teeth pendants recovered in the same layer are fully compatible (Fig 6L and 6M). Still, this mineral could also have been used for decorative purposes, directly penetrating the pendants themselves or the clothes to which they were attached.

### Use-wear

Similar use-wear marks were observed on eleven pendants, whether canines and incisor teeth or perforated bone pendants. The marks consist of polish around the edge of the hole, producing a slight modification of the original perforation shape (Fig 6G, 6H, 6L and 6M). Polish erased the striations generated during perforation by rotation, still observed in the unpolished areas. Eight of the teeth and bone perforated specimens from Dzudzuana and Satsurblia exhibit breakages at the edge of the hole where traction forces of a suspended item act (Fig 6N–6P). The location of these breakages and its association with polished perforations at the breaking point suggest they may be functional breakages [93].

Although it is commonly assumed that the perforated teeth and bone beads were used as personal ornaments, the specifics of their use are far from clear. The ethnographic record provides us with numerous examples of variegated utilization of suspended or attached objects, not as body ornaments but rather as clothing appendages, basket and bag accessories, etc. [45]. In the absence of a precise context (for instance a burial) associated with the teeth pendants of Dzudzuana and Satsurblia, we must be prudent when considering the functional use of the items. Nevertheless, technological studies of UP specimens from Eurasian and African records [44, 45, 93, 94, 101, 114, 115], as well as experimental work [99, 116], advocate the hypothesis that the perforated teeth and bones of Dzudzuana and Satsurblia are indeed pendants (namely personal ornaments). Accruement of polish inside the holes of the pendants, which had modified the original shape of the hole, suggests that the piece was worn, suspended, or sewn on clothing. Indeed, a relatively long wear of at least several months seems to have been needed to produce such a polish [99]. Moreover, the breakages at the distal part of the holes observed in several specimens are consistent with functional fractures typically documented in personal ornaments both archaeological or experimental [93]. Intensive use of the pendants indicates that they had a ‘long life’, and we can thus assume that these elements must have been of value to their owners.

## Discussion

Personal ornaments are among the cultural features considered as reliable proxies for the emergence of symbolically mediated behaviour (SMB) and complex societies [34, 37–39, 42, 55–58]. This assumption does not detract from the fact that aesthetics had most probably also played a role in the production of personal ornaments, as can be observed in extant primeval and complex modern societies.

In the Caucasus, the technical and symbolic use of bone, antler, teeth and shell seem to appear abruptly at the onset of the UP–from ca. 39 Ka (or even earlier and see above) years ago onwards [7, 10, 13]. Just like the chipped-stone techno-typology of the local EUP, also the symbolic sphere of the Caucasian hunter-gatherer groups, expressed in their personal ornaments, suggests some links with the coeval UP from other areas of Eurasia.

It is not easy to precisely characterize the production of the UP personal ornaments in the Caucasus overall through its technical aspects. This is due to the lack of technical studies of large corpora in the relevant regions; the flexibility in the techniques employed: and the limited sample size of the so far known assemblages. Contrary to the presence of bone and antler tools, personal ornaments are documented in the pre-LGM archaeological record of the Caucasus only in a few sites and in small numbers. These are Mezmaiskaya in the north [7, 11], Dzudzuana and Satsurblia in the south [13, this paper], and the Armenian site of Agithu-3, where there are only perforated shells with no teeth or bone specimens [32].

No particular association was observed in Dzudzuana and Satsurblia between a type of pendant, its raw material and a precise production technique. We can thus assume that on the whole, the UP Caucasian groups have employed different techniques for the manufacture of the pendants, similar to those observed in the European and Levantine EUP and at the beginning of the UP in Central Asia. The methods employed in these areas were varied, showing technical flexibility with gouging and rotation extensively documented [7, 44, 45, 52, 94, 117, 118].

**Fig 7:**
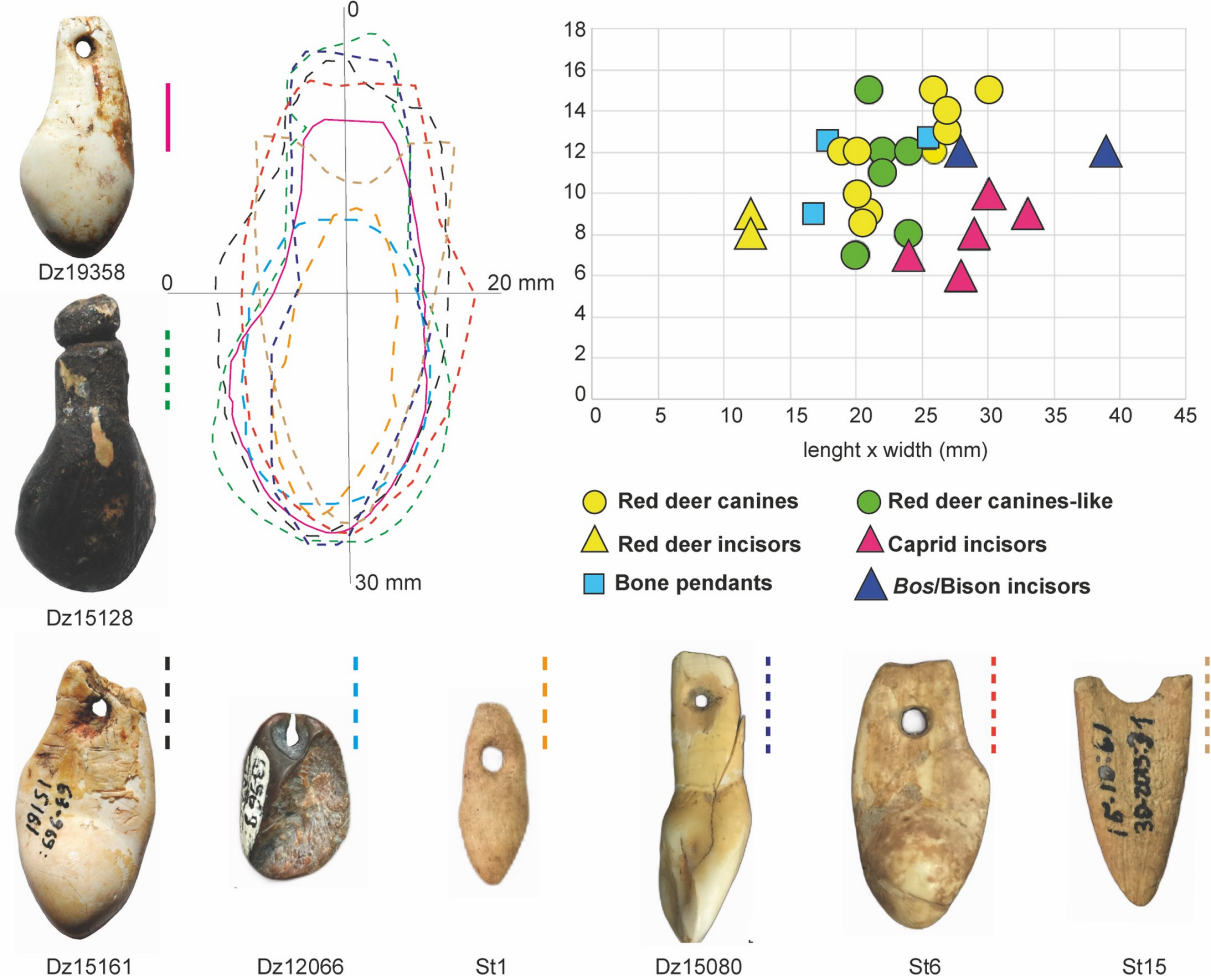
Morphometric comparison of pendants. Morphometrics of the pendants show the choice of a regular size and similar shape of both teeth and bone pendants.

Nevertheless, other behavioural aspects related to personal ornaments can provide us with new comparative elements. Selecting particular species and anatomical parts has demonstrated both shared and divergent choices regarding the aesthetic–cum-symbolic set–personal ornaments and decorated bones–from Western Europe and the Levant in the Early UP [51, 52]. In this regard, red deer vestigial canines seem to have a special status among the UP societies of the Caucasus, a feature shared with its counterpart groups in the Levant and Western and Central Europe as well as those from Central and Northern Asia [45, 52, 101, 119]. This can also be because the vestigial canines enter the chewing process only marginally and remain largely not worn during the animal’s life, which makes them appear particularly uniform in their shape and symmetrical, adding to those teeth further value, i.e., ‘pleasing to the eye’ [55–58]. Besides the ten red deer vestigial canines analysed, six other specimens (one red deer incisor and five bone pendants) were modified imitating their shape and size (Fig 7). Thus, sixteen of the full set of studied pendants–more than half of the assemblage—are red deer vestigial canines whether real or fake. Interestingly, in Mezmaiskaya, four of the seven perforated teeth recorded from EUP (1C), and late UP layers (1A2, 1A1) are red deer vestigial canines, with two caprid incisors and one perforated bone imitating them in shape and dimensions [7, 11].

**Fig 8:**
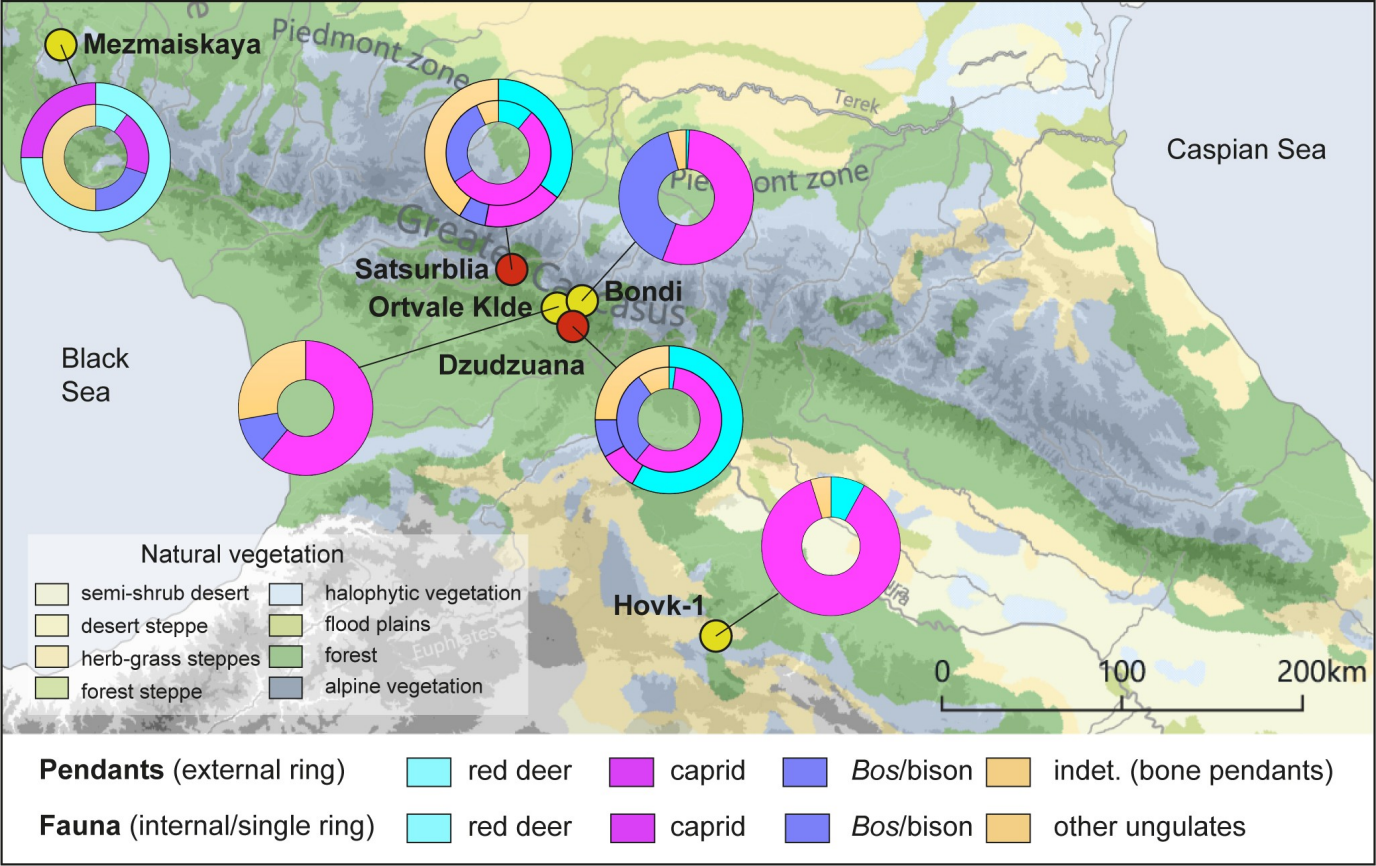
Map of the Caucasus with UP sites and distribution of raw material of the pendants. Dzudzuana, Satsurblia, Mezmaiskaya (external rings), and faunal remains (internal rings). Faunal data after: [2, 11, 17–19, 27, 28]. Background map after Stone, T.A., and P. Schlesinger. 2003. RLC Vegetative Cover of the Former Soviet Union, 1990. ORNL DAAC, Oak Ridge, Tennessee, USA. This dataset is openly shared, without restriction, in accordance with the NASA Data and Information Policy.

The significance accorded to the red deer among the pre-LGM UP groups in the Caucasus contrasts with its low importance in their subsistence. For example, in Dzudzuana cave of the nine perforated teeth, seven are of red deer. However, Dzudzuana red deer specimens represent 2% of the identified species of ungulates. In Satsurblia, out of ten teeth pendants five are of red deer. The frequencies of red deer at this site comprise only ca. 30% of the NISP in each layer. Among the teeth in Dzudzuana, no canines other than the perforated specimens were identified [25]. In Satsurblia only one canine was recovered, besides those shaped as pendants. The same is true for Mesmaiskaya where mammals assigned to size 2 (caprids) and size 4 (*Bos*/bison) represent ca. 40% of the NISP in layers 1C and 1A while red deer presence is much more restricted [11] (Fig 8).

The preference of one taxon and a particular tooth, namely the red deer vestigial canines, to make personal ornaments is a behavior shared by European, Levantine and Caucasian early UP groups. Perforated vestigial deer canines have been recently reported from Early UP in the Altaï mountains, in Central Asia e.g., Strashnaya [120], and Denisova [101], as well as from the North of China in Zhoukoudian Upper Cave [121]. Though the European hunter-gatherers exploited a large variety of teeth of diverse mammals, it appears that pierced red deer teeth were of importance and were worn by individuals traveling over long distances [122]. The apparent dissociation between animals whose meat was the staple food and animals whose teeth were used for ornaments is particularly marked in the case of red deer. The geographic distribution of red deer pendants in the European UP does not reflect the animals’ geographic distribution, because the pendants seem to have moved prior to the LGM beyond the extent of the red deer primary distribution areas [123, 124].

*Cervus elaphus* is one of the most abundant large mammal taxa in the European Late Pleistocene archaeological record [125, 126]. Although the red deer is an ecologically flexible species, it has been best adapted to temperate climate conditions and forested habitats [124]. Also in the Caucasus, red deer was present in EUP contexts [127]. Nevertheless, its frequencies in the archaeological record are anecdotal compared with that of caprids and, to a lesser extent, that of big bovids (*Bos*/Bison). According to currently available data, the latter faunal categories are the most common mammal species hunted and consumed in Caucasian UP sites, i.e., at Dzudzuana, Satsurblia, Mezmaiskaya, Ortvale Klde, Bondi Cave, and Hovk-1 [2, 11, 17–19, 27, 28] (Fig 8).

Apparently, prehistoric Eurasian societies had indeed a strong preference for the use of red deer canines as personal ornaments. Their presence in many prehistoric settlements, in different contexts, including single burials with dozens of those pendants in the UP [128], and in the Mesolithic [116, 129] as well as in extant ethnographic examples [130]–suggests that they possessed besides their aesthetic merit also a symbolic value for both Palaeolithic and post- Palaeolithic societies. Countless examples have been ethnographically observed of a particular animal special status within the symbolic world of extant hunter-gatherer societies [55–58, 131–133], suggested also for contexts from the Middle Palaeolithic [134] through the Mesolithic [135]. Yet, in most cases, these animals had seemingly an important nutritional value [136, 137] contra that of the red deer.

Still, the choice of C. *elaphus* vestigial canines could also be explained by the tooth’s particular rounded shape as well as its gloss and its tactile qualities [45]. Perhaps these were the reason for their imitation in different raw materials (bone and antler) by Palaeolithic hunter-gatherer groups in all Eurasian regions [7, 46, 107]. Similar qualities have been alleged for other raw materials used for ornaments like ivory, amber, and shells [45, 49, 138–141].

While it is tempting to argue for differences in the personal ornaments of the Caucasus between the EUP and the following UP assemblages, we should be cautious because the sample is relatively small. A shift has been evoked in the production of pendants at Mezmaiskaya. The pendants recovered from EUP layers (1C, 1B) were manufactured from a single species of teeth (Caucasian tur), as opposed to those from UP layers (1A1, 1A2) mainly made on red deer vestigial canines [7]. Personal ornaments also seem to be more common in later that in early UP layers, suggesting an increase of its importance from early to more recent UP phases. Still, such a scenario could be biased because in Dzudzuana for example, only a small part of the potentially occupied site surface was excavated [13]. Moreover, in Satsurblia the archaeological research is still ongoing, and thus we lack a complete picture of the site occupations precluding a definite comparison between early and later UP occupations [17].

The presence, at least in Satsurblia, of red deer teeth pendants from the early phases of the UP occupation on site appear to share behaviours in the symbolic sphere of the Caucasus UP societies with their counterpart groups in the Levant [142, 143].

The significance of red deer within the symbolic world of the Caucasian UP inhabitants of Dzudzuana and Satsurblia, in spite of their relative low abundance in the faunal remains when compared with other mammals (i.e., *Capra caucasica*, *Bos*/Bison) as in Mezmaiskaya, supports contacts and exchanges between North and South Caucasus in the late Pleistocene, already observed through the characteristics of the lithic assemblages. Also, identical techniques–mainly scraping, bifacial gouging and rotation–were employed by both, the Northern and Southern Caucasian UP groups to produce personal ornaments, although these techniques have been demonstrated to show certain flexibility, also observed in the early UP from other Eurasian areas. The technical choices and, especially, the taxonomic selection to produce the personal ornaments could imply the existence of cultural bonds between the Caucasian, Levantine and European hunter-gatherer groups at the early stages of the UP, considering the wide spread of this behavior all over Eurasia.

The data we present herein adds a significant aspect to the symbolic sphere of the Caucasus Palaeolithic societies, furthering our knowledge of late Pleistocene humans in the region. Recent studies have highlighted the importance of ornaments, be they perforated shells, or bone and teeth beads/pendants [48, 144–147]. It is now a given that understanding technological behaviors reflected in bone-implements and ornaments manufacture can greatly contribute to the study of the emergence and diffusion of Eurasian UP techno-cultural entities as well as their intra-actions and interactions, locally and globally [51, 52, 72, 148]. In the Caucasus, although the EUP lithic assemblages resemble more the contemporaneous Levantine Ahmarian than the European Early Aurignacian and the ‘Classic’ Levantine Aurignacian [7, 13, 14], the manufacture of the pendants and the taxa selected is closer to those from the EUP of the Levant. Bone implements are well represented in most of the Northern and Southern sites of the Caucasus. They will also further contribute, together with personal ornaments, to the refining of our knowledge as regards the techno-typological and conceptual behaviours of the last hunter-gatherers of the region, likely more complex than expected, and will highlight regional and trans-regional population and/or ideas movements.
